# Changes in respiratory structure and function after traumatic cervical spinal cord injury: observations from spinal cord and brain

**DOI:** 10.3389/fneur.2023.1251833

**Published:** 2023-10-06

**Authors:** Yongqi Xie, Liang Zhang, Shuang Guo, Run Peng, Huiming Gong, Mingliang Yang

**Affiliations:** ^1^School of Rehabilitation Medicine, Capital Medical University, Beijing, China; ^2^Department of Rehabilitation, Guangdong Provincial People's Hospital, Guangdong Academy of Medical Sciences, Guangzhou, China; ^3^Department of Spinal and Neural Functional Reconstruction, China Rehabilitation Research Center, Beijing, China; ^4^Center of Neural Injury and Repair, Beijing Institute for Brain Disorders, Beijing, China; ^5^Beijing Key Laboratory of Neural Injury and Rehabilitation, Beijing, China

**Keywords:** cervical spinal cord injury (CSCI), breathing, neuroplasticity, brainstem, bulbospinal pathway, magnetic resonance imaging, neuroimaging

## Abstract

Respiratory difficulties and mortality following severe cervical spinal cord injury (CSCI) result primarily from malfunctions of respiratory pathways and the paralyzed diaphragm. Nonetheless, individuals with CSCI can experience partial recovery of respiratory function through respiratory neuroplasticity. For decades, researchers have revealed the potential mechanism of respiratory nerve plasticity after CSCI, and have made progress in tissue healing and functional recovery. While most existing studies on respiratory plasticity after spinal cord injuries have focused on the cervical spinal cord, there is a paucity of research on respiratory-related brain structures following such injuries. Given the interconnectedness of the spinal cord and the brain, traumatic changes to the former can also impact the latter. Consequently, are there other potential therapeutic targets to consider? This review introduces the anatomy and physiology of typical respiratory centers, explores alterations in respiratory function following spinal cord injuries, and delves into the structural foundations of modified respiratory function in patients with CSCI. Additionally, we propose that magnetic resonance neuroimaging holds promise in the study of respiratory function post-CSCI. By studying respiratory plasticity in the brain and spinal cord after CSCI, we hope to guide future clinical work.

## 1. Introduction

**Figure 1:**
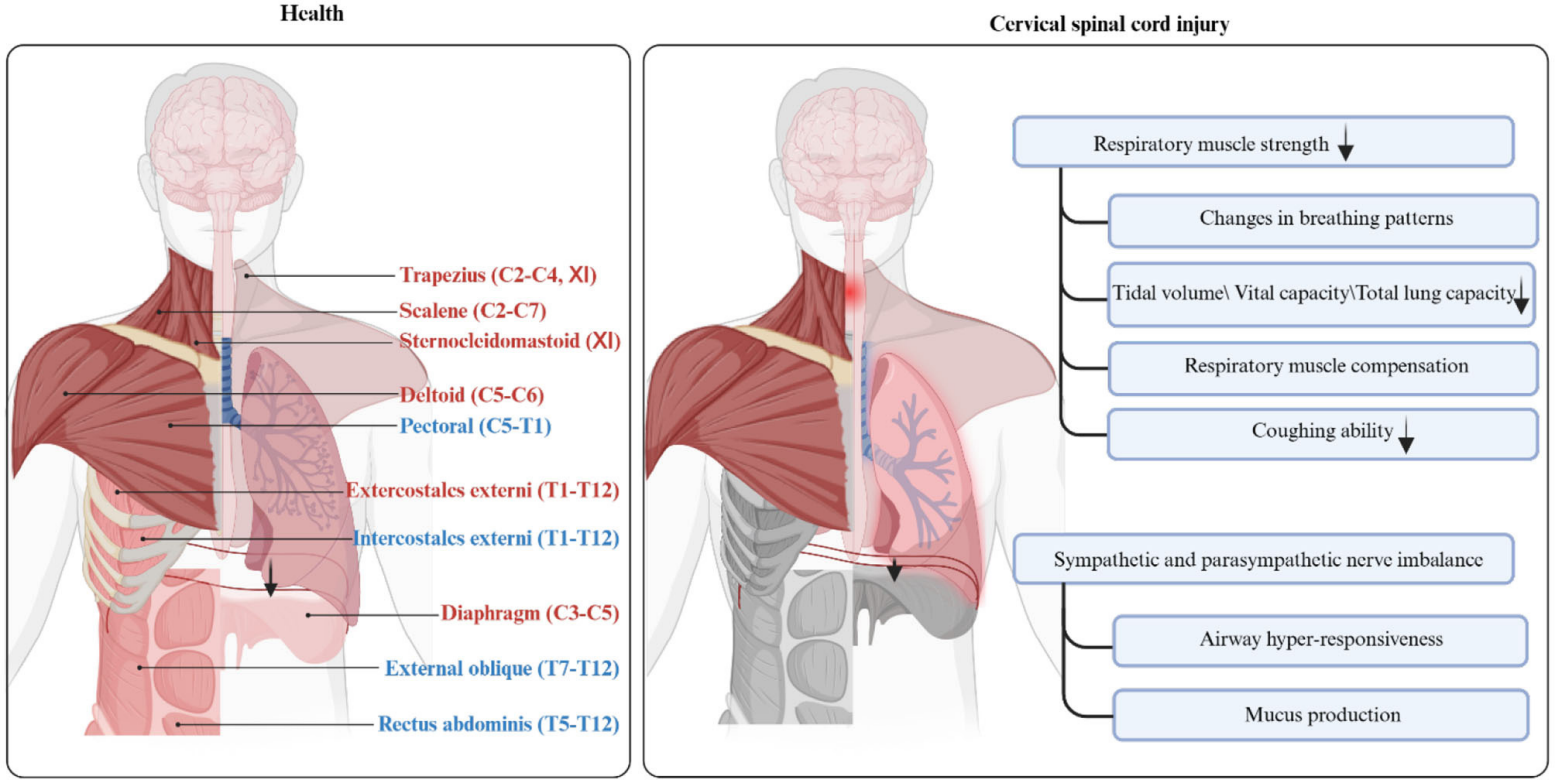
Anatomy and function of respiratory muscles in health and patients with cervical spinal cord injury. This figure shows the anatomical location and innervation of respiratory-related muscles. The inspiratory muscles are highlighted in red font, while the expiratory muscles are highlighted in blue font. Respiratory muscles that may be paralyzed after cervical cord injury are highlighted in gray. Created with BioRender.com.

Traumatic spinal cord injury (SCI) is an irreversible central nervous system disease with a high incidence rate of 50 per million individuals in China, with a higher incidence of cervical spinal cord injury (CSCI) at between 55.7% and 64.49% (1). Similarly, the incidence of SCI in the United States is 25–59 per million individuals (2, 3). Different degrees of respiratory dysfunction occur in patients with CSCI at different injury levels and degrees of injury and are mainly expressed as restrictive ventilatory deficits (4, 5). In addition, the degree of respiratory dysfunction following CSCI is compounded by an imbalance between the sympathetic and parasympathetic nervous systems. Parasympathetic dominance causes airway hyper-responsiveness and increased mucus production (Figure 1) (6). Consequently, respiratory muscles such as the diaphragm, intercostal muscles, and abdominal muscles become paralyzed, resulting in reduced inspiratory and expiratory forces, as well as a diminished ability to cough up secretions. During the acute phase, severe injuries often lead to respiratory complications including pneumonia, atelectasis, hypercarbia, hypoxemia, and potentially even death (7–9). Patients with milder impairments may experience changes in vocal quality or duration (10). Sleep apnea is also common among individuals with CSCI, although the exact underlying cause is still not fully understood (11, 12).

To maintain ventilation after spinal cord injury, patients experience shallow and fast breathing (13). Simultaneous paralysis of the respiratory muscles may prompt compensation of the neck muscles (14). Regardless of the initial defect, improvement in lung function will occur within 6 months of injury (15). These improvements can be attributed to biomechanical adaptations, neuroplasticity, and rehabilitation interventions (16). Breathing is a rhythmic process that involves the generation of respiratory rhythms by the brainstem respiratory centers, with contributions from higher brain centers in respiratory control (17, 18). Neuroplasticity refers to the continuous changes in the morphology and/or function of the neural control system based on experience (19). Preclinical research has shown that changes in neurons (20), activation of the cross-phrenic pathway (21–23), and axonal regeneration (24) play crucial roles in functional recovery following CSCI.

As the spinal cord and brain are interconnected, alterations in respiratory function due to CSCI can also affect the higher respiratory centers in the brain. Recent advancements in non-invasive neuroimaging techniques have enabled the examination of changes in respiratory-related structures and functions in the brainstem and subcortical layers of patients with CSCI. This provides valuable insight into the mechanisms underlying respiratory plasticity (25, 26). Neuroimaging can also assist in clinical decision-making by assessing respiratory function in patients with CSCI.

This review aims to explore the anatomy and function of the principal respiratory centers, discuss changes in respiratory function observed in clinical research on individuals with CSCI, examine findings from preclinical research regarding the structural basis for altered respiratory center function following cervical cord damage, and propose the potential use of neuroimaging to study the structure and function of respiratory centers in individuals with CSCI.

## 2. Anatomic basis of neural control of respiration

**Figure 2:**
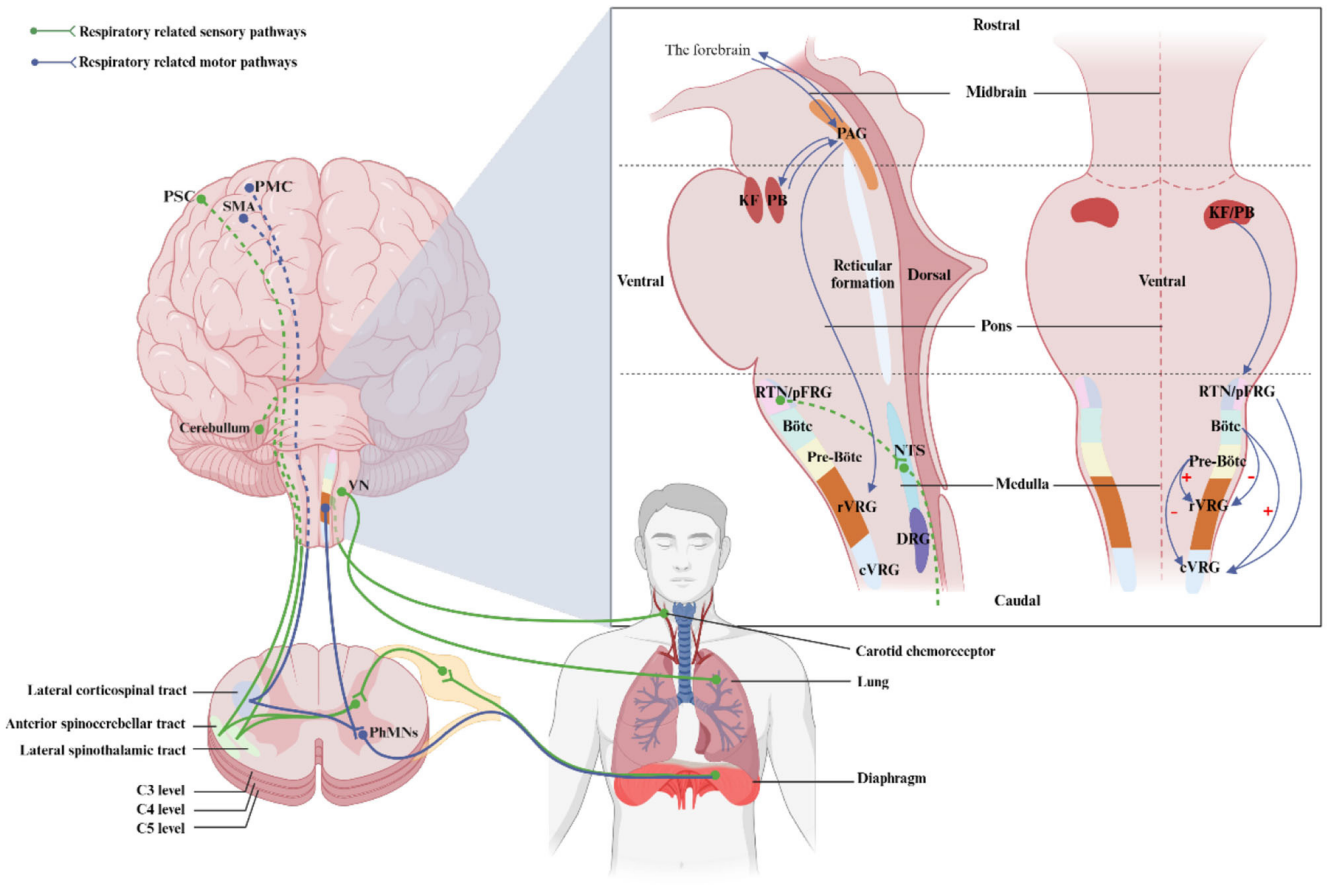
Location of regions and respiratory pathways involved in the control of breathing. This illustration shows the location of respiratory nuclei in the brainstem from a side and front view, as well as respiratory nerve pathways in a sagittal section. PMC, primary motor cortex; SMA, supplementary motor area; PSC, primary sensory cortex; rVRG, rostral ventral respiratory group; SpINs, spinal interneurons; PhMNs, phrenic motorneurons; PAG, periaqueductal gray; KF, kölliker-Fuse nucleus; PB, parabrachial nuclei; RTN, retrotrapezoid nucleus; pFRG, parafacial respiratory group; cVRG, caudal ventral respiratory group; NTS, nucleus of the Solitary Tract; DRG, dorsal respiratory group; VN, vagus nucleus. Created with BioRender.com.

Breathing is a complex and rhythmic activity that requires coordination between the respiratory and nervous systems. This section elucidates the central architecture and pathways responsible for respiration, enhancing our comprehension of disrupted respiratory neural pathways and neuroplasticity following SCI. Figure 2 visually depicts the interconnection between these domains.

### 2.1. Brainstem respiratory central pattern generator

The neural drive to breathe results from the integration of brainstem respiratory central pattern generators (rCPG) and the respiratory-related cortical networks (27–29). The rCPG is a hierarchical functional region; respiratory motions may result from integration among numerous brainstem nuclei (30–33). The main brainstem-associated respiratory nuclei, listed in descending order, include the periaqueductal gray (PAG) (34, 35), pontine respiratory group (PRG) (29, 36), retrotrapezoid nucleus (RTN)/parafacial respiratory group (pFRG) (37), pre-Bötzinger complex (pre-Bötc), Bötzinger complex (Bötc), ventral lateral medulla oblongata respiratory neuron group, and the nucleus tractus solitaries (NTS) (38). These nuclei harbor diverse neuron types and neurotransmitters, enabling them to perform distinct respiratory functions, as outlined in Table 1.

**Table 1:** The location and function of respiratory nuclei in the brainstem.

**Brainstem**	**Nucleus**	**Neurons or neurotransmitters**	**Function**
Midbrain	PAG	Neurotransmitters: 5-HT and neurotensin	•Coordinates specific patterns of respiration. •Input or output information to the forebrain. •Contributes to arousal and control of REM sleep (34).
Pons	KF/PB	• Neurons: laryngeal post-inspiratory premotor neurons • Neurotransmitters: glutamate, glycine, GABA, acetylcholine, norepinephrine, serotonin	•Regulates vocal fold closure and controls upper airway opening in the inspiratory and expiratory phases of the transition (39). •Regulates breathing frequency and tidal volume by regulating the duration of the inspiratory discharge of the VRG (40). •Switching respiratory phases, a classical apnea center (41).
Medulla	RTN/pFRG	• Neurotransmitters: VGlut2, Phox2b and NK1R • Neurons: oscillatory neurons	•Receives information from the center and peripheral chemoreceptors and inputs VRG (37, 42). •Delivers exhalation drive to cVRG.
	pre-Bötc	• Neurons: inspiratory neurons • Neurotransmitters: glutamate, glycine, GABA	•Respiratory rhythm oscillator. •Inhibits expiratory activity.
	Bötc	• Neurons: expiratory neurons • Neurotransmitters: glycine, GABA	•Respiratory rhythm oscillator. •Inhibits inspiratory activity.
	rVRG	• Neurons: bulbospinal premotor inspiratory neurons	•Transmits the inspiratory and expiratory drive to PhMNs.
	cVRG	• Neurons: excitatory bulbospinal expiratory neurons	•Transmit exhalation drive
	DRG		•Only drives inspiratory drive to PhMNs.
	RN	• Neurons: serotonergic neurons • Neurotransmitters: serotonin and co-localized peptides	•Participates in chemosensory regulation of breathing and stabilizes breathing (43).
	NTS	• Neurotransmitters: amino acids, biogenic amines, purines and peptides	•Coordinates respiratory and sympathetic responses to hypoxia.

PAG, periaqueductal gray; KF, kölliker-Fuse nucleus; PB, parabrachial nuclei; RTN, retrotrapezoid nucleus; pFRG, parafacial respiratory group; pre-Bötc, Pre Bötzinger; Bötc, Bötzinger; rVRG, rostral ventral respiratory group; PhMNs, phrenic motor neurons; cVRG, caudal ventral respiratory group; DRG, caudal respiratory group; RN, Raphe nucleus; NTS, nucleus of the Solitary Tract.

The respiratory rhythm originates from the ventral part of the medulla, specifically the pre-Bötc and Bötc. The pre-Bötc assumes the role of providing rhythmic excitation drive for respiration, while the Bötc engenders alternating inspiratory and expiratory patterns during normal breathing (44). The respiratory drives are conveyed to the ventral (VRG) and dorsal respiratory neuron groups (DRG) of the medulla oblongata (39). The rostral VRG is the largest group of inspiratory bulbospinal neurons and receives inspiratory drive from pre-Bötc neurons while being inhibited by expiratory Bötc neurons (39, 45). The rVRG transmits respiratory drive to phrenic motorneurons (PhMNs) via the bulbospinal respiratory neural pathway (46). The caudal VRG receives converging inputs, including those from the RTN/pFRG and BötC (39). The pons is proposed to interact with the medullary respiratory system (47). Respiratory adjustment primarily occurs in the midbrain, pons, and related cortical regions. The PAG mainly regulated the patterns of respiratory and contributed to arousal and control of sleep (34). Respiratory adjustments occur mainly in the PRG and the facial nucleus. The PRG, consisting of the Kölliker-Fuse nucleus (KF) and the parabrachial nuclei (PB), project upward to the amygdala and hypothalamus (48) and downward to the VRG through medullary raphe neurons (49). The RTN/pFRG serves as a conditioned oscillator of expiratory activity and serves as a significant generator of inspiratory rhythms (50, 51). Additionally, the lateral reticular nucleus also affects respiratory control and arousal (52).

### 2.2. Respiratory-related forebrain regions

Advanced respiratory centers connected to breathing include the cerebral cortex, thalamus, hypothalamus, hippocampus, extended amygdala, and limbic system (53–55). For details, please refer to this literature (29). Initial indications of a direct connection between the cortex and motor neurons were observed when the primary motor cortex was stimulated, inducing activation of the phrenic nerve in cats (56). Subsequently, the relationship between spontaneous breathing and the primary motor cortex was confirmed using transcranial electrical or magnetic cortical stimulation (57–60) and positron emission tomography (61). Furthermore, evidence suggests the existence of a rapid pathway from the supplementary motor area (SMA) to the diaphragm, with the SMA exerting a more pronounced excitatory effect on the diaphragm (62). The premotor area was thought to be involved in respiratory control during articulation (63). While our understanding of the brainstem respiratory center has advanced (39), the control mechanisms of the higher respiratory centers and their performance under varying conditions remain unclear.

### 2.3. Respiratory afferent and efferent pathways

#### 2.3.1. Respiratory afferent pathways

Respiratory information is collected through chemoreceptors, bronchial and pulmonary receptors, and respiratory muscle proprioceptors (Figure 2). Chemoreceptors detect CO2, O2, and pH levels, and transmit this information to the central chemical-sensitive neurons in the brainstem (37, 64). Sensory information from the lungs and the airways is conveyed via the vagus nerve, which serves as the link between the lung and brain (65, 66). Besides, the proprioceptive information from the respiratory muscle is sent to the cerebellum through the spinocerebellar tract for processing (27). The respiratory sensory information is also projected to supraspinal structures such as the hypothalamus, thalamus, cingulate gyrus, and sensory cortex, where it is integrated and further processed to regulate breathing (48, 67, 68).

#### 2.3.2. Respiratory efferent pathways

Respiratory movements are facilitated by transmitting autonomous respiratory impulses from the rhythm-generating respiratory center and the reticular structure via the bulbar spinal cord pathway. Additionally, voluntary respiratory impulses from the respiratory-related cortex are transmitted to the respiratory-related skeletal muscles through the corticospinal lateral tract (27). The respiratory muscles can be categorized into upper respiratory muscles and trunk respiratory muscles. The upper respiratory muscles are critical to maintaining upper respiratory tract patency and airway pressure (69). The abnormalities in upper respiratory muscles may cause sleep apnea and dysphagia (11, 70, 71). Trunk respiratory muscles are closely involved in lung ventilation. The diaphragm is the primary inspiratory muscle, contributing to approximately 65% of tidal volume during calm breathing (28). Contraction of the abdominal muscles and internal intercostal muscles helps the diaphragm return to its resting position, reduces intra-abdominal pressure, and facilitates expiration (72, 73).

## 3. Structural basis of respiratory plasticity after CSCI

### 3.1. Neuronal changes

#### 3.1.1. Respiratory motor neuron

The phrenic motor neurons and the bulbospinal nerve pathway are directly affected following C2 hemisection, leading to a reduction in tidal volume due to decreased respiratory drive to the phrenic neurons (4). In the mid-cervical spinal cord contusion mice model (74) and the unilateral C4 contusion rat model (75), the same ipsilateral PhMNs can be lost within 24 h, accompanied by phrenic nerve axon deformation and denervation of the phrenic neuromuscular junction, resulting in impaired diaphragm function. Despite increasing lesion volume, there is minimal progression of the injury over an extended period (75). During the chronic phase, PhMNs of Sprague-Dawley (SD) rats tend to return to control values, potentially due to the influence of brainstem upper respiratory neurons on early PhMNs function (76). Additionally, intercostal muscle neurons are also affected by damage to the bulbospinal pathway, with recovery similar to PhMNs (77). Intercostal muscle recovery is observed in both spinal cord hemisection (77) and contusion SD rats models (78). All of the above models of CSCI confirm that respiratory motor neurons are immediately damaged, resulting in varying levels of muscle paralysis. However, with time, the paralyzed muscles all show functional recovery, which may result from replacement with other neurons or lateral bypass repair.

#### 3.1.2. Spinal interneurons

The location of Spinal interneurons (SpINs) are distributed in the dorsal horn, around the central canal, in the gray matter of the spinal cord (79). Morphological and electrophysiology evidence has confirmed that SpINs receive their excitatory inspiratory drive from the rVRG (80–82). With our understanding of respiratory neuromodulation, it appears that SpINs play a role in promoting respiratory recovery (20, 83). During acute hypoxia, a network of spinal cord interneurons becomes activated, facilitating synaptic connections from the contralateral side of the injury (84). In chronic spinal cord injury, SpIN recruitment increases along with increased interaction with the bulbospinal pathway, suggesting a potential role for SpINs in respiratory integration (85). At the same time, thoracic SpINs may serve as reliable neurons connecting intercostal neurons after disruption of the bulbospinal pathway (86). Spinal cord proprioceptive interneurons may also be involved in the reconstruction of respiratory function after thoracic segment SCI and play a key role in functional recovery following injury (87).

#### 3.1.3. Respiratory-related neurons in the brainstem and subcortical structures

Several studies have revealed that modifications occur in the brainstem and higher brain structures during the acute phase of CSCI that go beyond the plasticity of respiratory nerves within the spinal cord segment. These changes may arise from the presence of interconnections or unidirectional connections between the rRVG and other nerve nuclei (88–90). For instance, there is a tendency for an increase in rVRG expiratory activity while cVRG expiratory activity declines (89). Similar alterations in the reticular fiber structures have been observed following spinal cord hemisection, indicating their association with the restoration of respiratory function (89–91). Researchers have utilized transcranial magnetic stimulation in mice and rats to visualize respiratory plasticity after CSCI (92, 93). Vinit discovered ipsilateral diaphragmatic motor-evoked potentials when the coil center was positioned 6 mm caudal to the pons, suggesting that stimulated PAG might influence breathing (92). Although current investigations on the remodeling of respiratory nociceptors after CSCI primarily focus on fundamental research (13). It is important to acknowledge the differences between human structure and function when extrapolating findings from animal models (94). Section 6 describes the alterations in cortical structures related to respiration after SCI.

### 3.2. The cross-phrenic phenomenon

**Figure 3:**
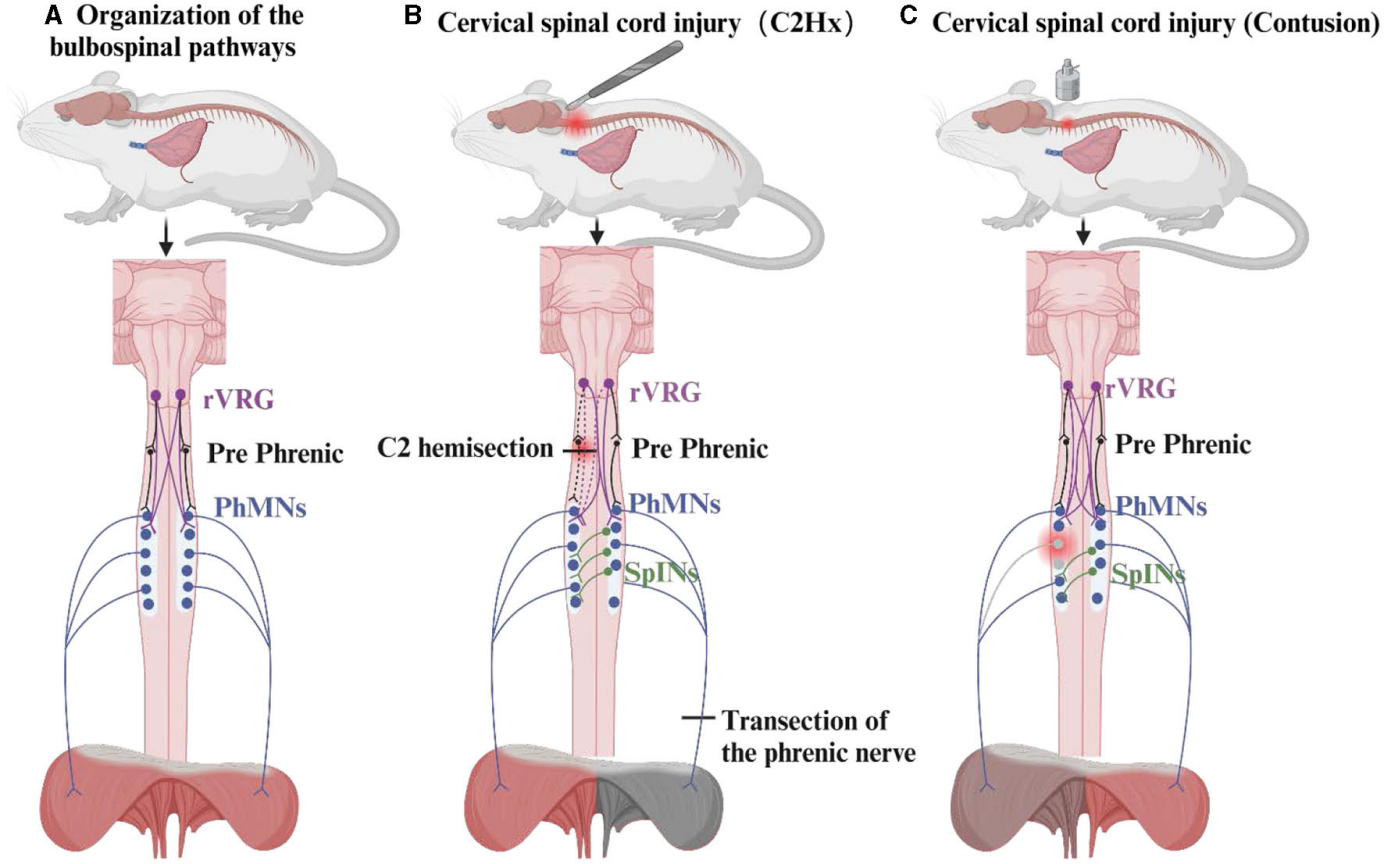
The organization of the bulbospinal pathways and the crossed phrenic phenomenon. The crossed phrenic phenomenon (CPP) of the C2 hemisection model and the contusion model are described here. **(A)** Composition of the respiratory pathway in the bulbospinal pathway. **(B)** CPP activated by the C2 hemisection model allows for recovery of the diaphragm on the side of the injury. **(C)** Activation of CPP in the cervical medullary contusion model resulted in mild damage to the diaphragm.

The strongest evidence that potential respiratory neural pathways are activated in the weeks or months following high CSCI is the crossed phrenic phenomenon (CPP). CPP was first discovered by John Porter in 1895 through the C2 hemisection model (21). Since then, the neuroplasticity of diaphragm neuromotor control after CSCI has been studied. Cervical medullary hemisection interrupts the inspiratory drive of the ipsilateral PhMNs, paralyzes the ipsilateral diaphragm, and then achieves functional recovery of the ipsilateral diaphragm by severing the contralateral phrenic nerve to induce CPP (21, 95). The occurrence of CPP serves as a respiratory-related stressor leading to modulation of respiratory function, and the bulbospinal pathway contralateral to the injury is activated to cross over at the level of the phrenic nucleus within the spinal cord to innervate the ipsilateral PhMNs (4). Similarly, in rats with unilateral CSCI, the “crossed-spinal” pathways are also observed in the ipsilateral intercostal activity (77). Further studies have identified CPP as a state-dependent phenomenon that is induced by certain respiratory stressors, such as contralateral phrenic nerve dissection, hypercapnia, hypoxia, and asphyxia (96, 97). One study has identified CPP within a few days of injury (98), while other studies identify CPP within 2 weeks of C2 hemisection (99, 100). Beyond the C2 hemisection model, researchers have developed alternative models, including a cold block of the high cervical segment, C1 hemisection with the decerebrate brain, C2 hemisection with injury of the ventral lateral aspect, C4 hemisection, and contusion of the middle and high cervical segments, to replicate the plasticity of respiratory function observed in human SCI (Figure 3) (96). Recently, Vinit et al. demonstrated the presence of CPP by applying Transcranial Magnetic Stimulation (TMS) 1 h after the C2 injury, which resulted in evoked potentials in the diaphragm on the injured side (93).

### 3.3. Axonal regeneration

The regenerative capacity of axons is influenced by the distance between the injury site and the cell body. In the case of CSCI, the corticospinal tract has a lower potential for axonal regeneration compared to the bulbospinal tract (45). Axon resection in the bulbospinal pathway, resulting from C2 lateral spinal cord injury in rats, leads to degenerative changes. However, it can also trigger regenerative processes such as lateral sprouting or medial axonal remobilization (101). The recruitment of Spinal Interneurons (SpINs) at the C1 level was found to be increased when interacting with the bulbospinal pathway (101). Additionally, other substances have been discovered to promote axonal regeneration related to respiration. Phosphatase and tensin homologs promote substantial long-distance regeneration of rVRG axons after hemisection (102–104), but have less ability to restore diaphragm function. Chondroitinase ABC (ChABC) inhibits the up-regulation of chondroitin sulfate proteoglycans around phrenic motor neurons after spinal cord injury (24). When combined with autologous nerve grafts, ChABC restores functionality in paralyzed diaphragms (24). Consequently, the activation of SpINs and the use of axonal growth-promoting drugs are potential therapeutic strategies. However, the extent of axonal regeneration necessary for functional recovery remains largely unknown in most cases (105).

Further research is required to investigate supraspinal neurons and SpINs following CSCI, including studies on temporal alterations, impacts on diaphragm movement, and performance in various injury models.

## 4. Changes in respiratory function after CSCI

The coordination between the spinal cord, brainstem, and cerebral cortex is essential for the process of breathing. However, respiratory afferent and efferent pathways may be partially or completely disrupted following CSCI, leading to respiratory dysfunction. Figure 1 illustrates the altered respiratory function following CSCI.

### 4.1. Changes in breathing patterns

Following CSCI, patients may experience shallow and rapid breathing to maintain minute ventilation, but tidal volume tends to decrease under these circumstances (4, 100). Paralysis of the diaphragm due to SCI can result in defective breathing patterns such as paradoxical movements (106). During inspiration, the paralyzed diaphragm moves upward, while the abdomen moves inward as the accessory inspiratory muscles lift and the rib cage simultaneously expands, lowering intrathoracic pressure. This condition is more pronounced in the supine position.

### 4.2. Compensation of respiratory muscles

Damage to phrenic neurons after SCI can induce the expression of proteases and atrophy-related genes, leading to immediate atrophy of all types of diaphragm fibers (5). Moreover, early use of a ventilator can accelerate the atrophy of the diaphragm (107), and diaphragm contractility can decrease by up to 40% within 8 weeks after injury (108). Following SCI, compensatory hypertrophy of the diaphragm occurs due to a reduction in the diaphragmatic contraction rate (109). However, individuals of Chinese tend to exhibit less diaphragmatic hypertrophy than individuals of European or American descent (110). Accessory muscles such as the deltoid and trapezius play a crucial role in respiratory function following CSCI (14). In addition, the abdominal muscles are paralyzed and other muscles such as the latissimus dorsi and pectoralis major are activated during coughing (111). During respiratory muscle strength tests, the rhomboid muscle is more involved during the maximum inspiratory pressure test and the pectoralis major and latissimus dorsi are more involved during the maximum expiratory pressure test, compared to non-injured individuals (112).

### 4.3. Changes in pulmonary function

Pulmonary function often significantly improves within the initial 6-month period after injury and less so thereafter (113). Multiple factors, including the level, degree, and timing of injury, age, body position, and obesity, can influence changes in pulmonary function after CSCI (114, 115). Early improvements in respiratory function after SCI may be due to early edema subsidence, compensatory respiratory strategies, and altered respiratory muscle biomechanics. In contrast, for patients with chronic CSCI, age, persistent wheezing, and obesity are important factors affecting lung function. However, the degree and level of injury may not be one of the factors affecting pulmonary function in chronic spinal cord injury (116). A long-term follow-up found that age (>30 years) and BMI (>30 kg/m^2^) as important factors affecting lung function after CSCI (117). A retrospective study of 339 patients with CSCI revealed that smoking, persistent wheezing, obesity, and MIP, as well as SCI levels and integrity, were significant determinants of lung function (118).

### 4.4. Increased frequency of sleep apnea

Although there are different criteria for evaluating sleep breathing disorders, the incidence of sleep apnea in patients with SCI was higher than in the healthy population, and the incidence of patients with tetraplegia was higher than in the paraplegic population (119, 120). The exact mechanism underlying sleep apnea after CSCI remains unclear, although some researchers have suggested associations with upper airway muscle inactivation, autonomic dysregulation, reduced lung volume, and modifications in brainstem plasticity or chemosensitivity (11, 121).

## 5. Magnetic resonance neuroimaging in respiratory structure and function after CSCI

In clinical practice, pulmonary function tests, chest computed tomography, chest fluoroscopy, chest radiography, diaphragmatic ultrasound, diaphragmatic electromyography, and phrenic nerve stimulation are used to evaluate the lung function of patients with CSCI (110, 122, 123). However, these methods only offer localized information on respiratory function and do not serve as a benchmark for changes in spinal cord and respiratory center structures and functions. In recent times, nuclear magnetic imaging methods such as magnetic resonance imaging (MRI), functional magnetic resonance imaging (fMRI), and diffusion tensor imaging (DTI) have provided insights into the structure and function of the brain and spinal cord following SCI, enabling non-invasive visualization of the brainstem. These imaging techniques facilitate the evaluation of brain and spinal cord structure and function, prediction of neurological function, and assessment of treatment effectiveness (124, 125).

### 5.1. Structure MRI

Structural brain volume data can be obtained from T1-weighted images in MRI, and mathematical algorithms can be used to extract relevant brain features and perform statistical analysis of brain volume, morphology, and surface area (126). The reorganization of gray matter is caused by cell atrophy or apoptosis after axon transection, while the changes in white matter are caused by axon demyelination and deformation (127).

Following acute injury, the anatomy and organization of the spinal cord undergo significant changes. Several studies have shown that there is a reduction in the area and width of the cervical medulla, changes in the gray matter of the primary cortex and limbic system (50, 128), and a reduction in the dorsal pyramidal tract of the medulla oblongata and the white matter of the cerebellar peduncle (129) in the acute phase. These changes may result in clinical symptoms such as impairment of motor and sensory function.

In the subacute to chronic phase, atrophy and microstructural changes in the spinal cord proceed further. Progressive atrophy and microstructural changes occur in the thalamus, anterior cingulate gyrus, insula, pons, and secondary sensory cortex (130).

In the chronic phase after spinal cord injury, atrophy of the corticospinal tract and medial thalamic tract occurs, with reduced gray matter in the brainstem around the midbrain aqueduct, dorsal pons, and dorsal medulla oblongata myelin (131). A significant reduction in dorsal anterior spinal cord volume at the spinal medullary junction, is consistent with clinical histologic evidence (132). This may reflect the Wallerian degeneration of the associated axons after spinal cord injury. Extensive atrophy and microstructural changes are also observed in the cerebral tracts and sensory-motor cortical areas of the spinal cord at 2, 6, and 12 months after acute spinal cord injury (133). Furthermore, cortical gray and white matter volumes were reduced at the level of the medullary aqueducts compared to healthy controls at 12 months post-injury.

These findings suggest that structural changes in the spinal cord following acute spinal cord injury may be associated with respiratory dysfunction, particularly alterations in brainstem microstructure. However, further research is required to gain deeper insights into the mechanisms underlying these changes and their role in respiratory dysfunction.

### 5.2. Diffusion tensor imaging

Diffusion tensor imaging (DTI) is an imaging technique capable of quantifying atrophy, demyelination, and iron deposition within the spinal cord and cerebral cortex (134). It is widely used to assess spinal cord, brainstem, and brain alterations after CSCI (135–137). The diffusion of water molecules in neural tissues is mainly limited by cell membranes and myelin sheaths. When neurodegenerative lesions occur, the tissue produces more free water, leading to re-diffusion perpendicular to the white matter (124). Parameters commonly used in DTI include fractional anisotropy (FA), mean diffusivity (MD), and apparent diffusion coefficient (ADC). The definitions of these parameters can be found in more detail in the literature (124).

In the acute phase, a significant decrease in FA and an increase in MD at the level of injury can be observed after SCI, but changes in ADC are controversial (124, 138). Similarly, a decrease in FA values at the lesion level and in the upper cervical spinal cord can also be observed in the chronic phase (136, 139–142). A long-term follow-up study revealed worrisome results: gray matter below the C2/3 level decreased by 0.7% per month and white matter by 0.34% per month (143). At the level of the cerebral peduncle, individuals with cervical ASIA A/B SCI showed greater degrees of axonal damage and edema/tissue loss, but no statistical differences were found in whole-brain white matter compared to healthy individuals (144). Of concern is that spinal cord injuries also cause changes in the white matter of the brain. A DTI study found that chronic SCI has a wide range of neurodegenerative effects in the brain that are not limited to motor pathways (145). Meanwhile, patients with CSCI in the chronic phase continue to experience a slow decline in degenerative FA in the midbrain, pontine, and superior white matter of the medulla, while MD keeps increasing, probably because of cumulative cell membrane loss caused by delayed damage to glial cells or axons (146).

In addition, DTI can also be used to assess treatment efficacy. Gu et al. used DTI and immunohistochemistry to assess the efficacy of axonal regeneration, and DTI reflects the center of injury as well as the immediate condition of neural damage and the process of axonal regeneration (147). Zhang et al. used DTI to explore the efficacy of vocal therapy on respiratory function in patients with CSCI and found that neural networks related to respiration in the medulla and cortex became more active (148). To some extent, this reflects the treatment-facilitated recovery process in the brainstem and brain-related respiratory nerves. The combined application of DTI and fMRI enables central pathway monitoring and treatment evaluation (149).

In summary, scientific studies have provided valuable insights into the altered state of the spinal cord and brain following acute and chronic spinal cord injury. However, the understanding of these changes remains somewhat controversial, and further research is needed to explore the mechanisms and clinical implications in depth.

### 5.3. Functional MRI

The aforementioned techniques are primarily utilized to evaluate structural changes. It is equally crucial to assess the functional reorganization of the brain and spinal cord. While fMRI can detect alterations in blood oxygen level-dependent (BOLD) signals, the pattern of these changes is expected to be utilized in assessing the ability to reorganize function (125, 150).

Acute SCI patients have decreased functional connections between the bilateral primary sensory cortex and motor cortex, while functional connections increase between primary sensorimotor cortex, premotor cortex, supplementary motor area, thalamus, and cerebellum (151). Patients with advanced SCI may have changes in cognitive-related areas, such as increased functional connections between the dorsal anterior cingulate cortex and the motor cortex (152). Various studies have found that the classic somatosensory pathway degenerates (153) and reorganizes (154, 155) after SCI, meaning that the spinal thalamic tract or dorsal tract transmits sensory signals to the thalamus, which is transformed and projected to the primary sensory cortex. Others have found that sensory input in patients with incomplete CSCI may be mediated by alternative pathways, which may consist of the ipsilateral cerebellum, pons, and contralateral posterior central gyrus (127). In patients with incomplete spinal cord injury, the visual cortex supplements sensory and motor inputs (156). Many studies have found that the cerebellum is important for activation after SCI (157). The functional connections between the cerebellum and primary motor cortex, primary sensory cortex, and primary auditory cortex can be found in patients with complete injury (151, 152, 158). In patients with chronic CSCI, whole-brain network connectivity is reduced, and increased connectivity is observed in the subnetworks of the sensory-motor cortex and cerebellum (159). Although the cerebellum is not a region of respiration in the traditional sense, we speculate that the cerebellum may play an important role in respiratory sensory and motor function after SCI.

Respiration-related fMRI investigations primarily focus on the influence of subcortical structures. Studies have shown that respiratory control is connected to brainstem and subcortical activity, with many indicating the maintenance of connectivity between cortical and brainstem regions (53, 54, 160). Pattinson et al. identified regions of the brainstem and thalamus in healthy subjects that respond to CO_2_ stimulation by fMRI and diffusion fiber tracing. They explored a link between the thalamus and higher cortical tracts, suggesting that the thalamus can play a role in respiratory control, but the relationship between structure and function was not explored in this study (161). The brainstem could be studied by BOLD fMRI to observe characteristics of the respiratory center at rest and its corresponding mechanisms (162). There are fewer fMRI studies on respiration in patients with SCI, likely due to the challenging nature of conducting respiratory center fMRI owing to the unique anatomy and small size of the brainstem. Relevant atlases are lacking, and physiological noise from cerebrospinal fluid and arteries obscures measurements near the brainstem (150, 163, 164). Nonetheless, researchers have explored several methods for brainstem fMRI data processing (55, 164, 165).

## 6. Concluding remarks

Traumatic spinal cord injury is a devastating and irreversible trauma. Despite the current advancements in clinical assessment, treatment, and medical management, such as rehabilitative care, patients still suffer significant neurological impairments. The focus of current research on cervical spinal cord injury is the restoration of respiratory function. From basic clinical studies, most of them have focused on respiratory plasticity at the spinal cord level, revealing the neuroplasticity of the respiratory network after cervical cord injury. However, the respiratory plasticity of structures above the spinal cord remains incompletely understood. In recent years, the use of MRI in traumatic spinal cord injury has shifted from clinical assessment to neuroimaging biomarkers (125). MRI can elucidate the injury mechanism, assess its extent, and serve as a surrogate endpoint for clinical trials. Previous studies have shown that brain reorganization following SCI primarily occurs through compensatory sensory connections in the auditory or visual cortex and cerebellar function. Zhang et al. also demonstrated that vocal breathing training improved respiratory function more in CSCI patients (148). Further research is needed to explore whether increasing sensory inputs in the visual, auditory, and other pathways during respiratory function training can enhance therapeutic outcomes (25). Additionally, the role of the cerebellum, which has been neglected in the past, should be considered. However, the study of respiratory function in cervical cord injury is still in its early and exploratory stages, with many pressing challenges to address in future research. Firstly, large-scale follow-up studies are lacking to provide comprehensive insight into disease progression. Secondly, SCI patients may experience long-term complications such as spasticity and neuropathic pain, which may cause changes in brain function, emphasizing the need to remain focused on injury severity, time after injury, recovery time, and the effects of drug intervention (166). Thirdly, smaller anatomical changes in the brainstem cannot be detected after SCI. Lastly, magnetic resonance neuroimaging shows that the brain undergoes reorganization after SCI, but it remains to be explored whether these changes are beneficial or harmful (167).

Magnetic resonance neuroimaging is expected to provide relevant evidence for respiratory plasticity and targeted treatment for CSCI, but this may require interdisciplinary research in neuroimaging, nuclear medicine, rehabilitation medicine, and other fields. The shortcomings of current magnetic resonance neuroimaging must be addressed while indicating new directions for the clinical treatment of respiratory issues in CSCI patients.

## Author contributions

YX wrote the main manuscript text. LZ and RP completed extensive literature searches and reviews. The figures and tables were made by YX and SG. SG, HG, and MY did language modification. All authors contributed to the editorial process and approved the final version of the manuscript.
